# Balloon Fracture Fenestration after Frozen Elephant Trunk Deployment to Eliminate Retrograde False-Lumen Perfusion in a Type B Dissection–Complicated Arch Aneurysm

**DOI:** 10.70352/scrj.cr.26-0016

**Published:** 2026-04-08

**Authors:** Daichi Takagi, Gembu Yamaura, Kentaro Kiryu, Takeshi Arai, Itaru Igarashi, Yuya Yamazaki, Wataru Igarashi, Hiroyuki Nakajima

**Affiliations:** Department of Cardiovascular Surgery, Akita University Graduate School of Medicine, Akita, Akita, Japan

**Keywords:** aortic dissection, aortic aneurysm, thoracic endovascular aortic repair, frozen elephant trunk, total arch replacement, balloon fracture fenestration

## Abstract

**INTRODUCTION:**

Arch or proximal descending aortic aneurysms complicated by Stanford type B aortic dissection (TBAD) may require extensive open repair with an open proximal anastomosis. Although a staged modified elephant trunk strategy is a reasonable option, the need to undergo two major operations is burdensome. The frozen elephant trunk (FET) technique enables concomitant treatment of the descending aorta after arch replacement; however, persistent retrograde false-lumen perfusion may result in incomplete aneurysm exclusion and inadequate aortic remodeling. We report a single-stage approach using adjunctive balloon fracture fenestration after FET deployment.

**CASE PRESENTATION:**

A 78-year-old man was referred 63 days after TBAD onset. CT demonstrated a 60-mm enlargement of the proximal descending aorta immediately distal to the left subclavian artery, a 37-mm distal segment, and enlargement of the ascending aorta to 42 mm, making standard thoracic endovascular aortic repair unsuitable. Total arch replacement using FET was performed, followed by balloon expansion of the FET to fracture the dissection flap, after which angiography demonstrated resolution of the endoleak. At 6 months, CT revealed distal stent graft-induced new entry, along with aneurysm shrinkage, complete thrombosis, and no communication between the proximal and distal false lumens.

**CONCLUSIONS:**

Balloon fracture fenestration applied to total arch replacement with FET may interrupt retrograde false-lumen perfusion and enhance distal sealing in selected TBAD-associated arch or proximal descending aneurysms. However, careful vigilance is required to mitigate potential complications, including distal stent graft-induced new entry and spinal cord ischemia. This approach may mitigate a major limitation of staged repair, namely the risk of interval rupture while awaiting completion of the second-stage procedure.

## Abbreviations

DHCA
: deep hypothermic circulatory arrest
dSINE
: distal stent graft-induced new entry
FET
: frozen elephant trunk
FL
: false lumen
TAR
: total arch replacement
TBAD
: Stanford type B aortic dissection
TEVAR
: thoracic endovascular aortic repair
SCI
: spinal cord ischemia

## INTRODUCTION

In arch or proximal descending aortic aneurysms complicated by Stanford type B aortic dissection (TBAD), extensive aortic replacement with an open proximal anastomosis is often required and carries a risk of early mortality and major morbidity.^1)^ The two-stage modified elephant trunk approach offers a reasonable solution; however, the need to undergo two major operations is daunting for many patients.^2)^ Total arch replacement (TAR) with the frozen elephant trunk (FET) technique enables concomitant treatment of the descending aorta during arch replacement.^3)^ Nevertheless, persistent retrograde perfusion through the false lumen (FL) may maintain flow into the aneurysm, leading to incomplete aneurysm exclusion and inadequate aortic remodeling.^4)^ In this case, we sought to achieve complete aneurysm exclusion by performing adjunctive balloon fracture fenestration after FET deployment, with the aim of expanding the true lumen and enhancing distal sealing to interrupt retrograde FL perfusion in a single-stage procedure.

## CASE PRESENTATION

**Fig. 1:**
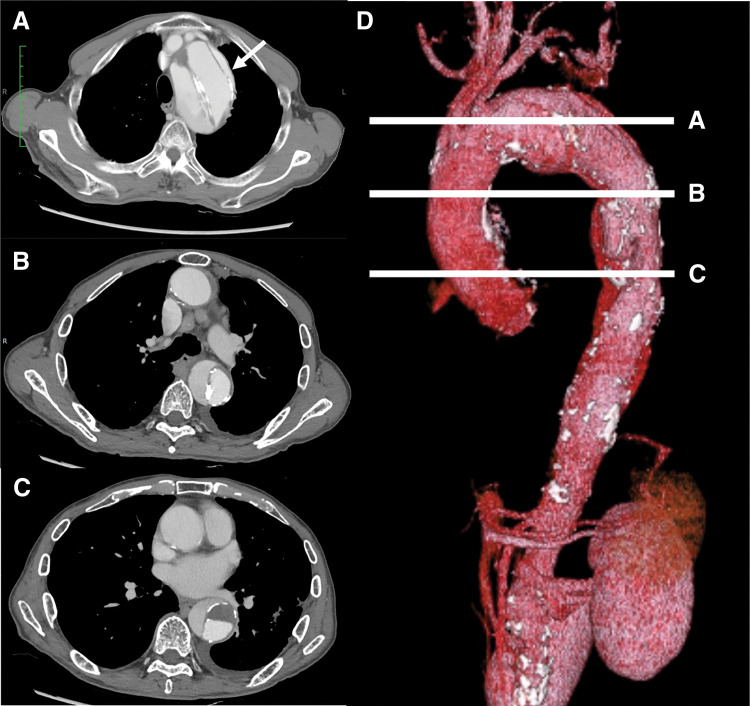
Preoperative CT of aortic arch and proximal descending aneurysm. CT axial images at the levels of the aortic arch and proximal descending aorta, bronchial bifurcation, and left atrium before surgery (panels **A**, **B**, and **C**, respectively). A 3D CT reconstruction before surgery is shown in panel **D**. White lines A, B, and C in panel **D** correspond to the axial images in panels **A**, **B**, and **C**, respectively.

Written informed consent was obtained from the patient for publication of this case report and the accompanying images. A 78-year-old man with an aortic arch aneurysm developed TBAD and was referred to our hospital for treatment 63 days later. CT (**Fig. 1**) demonstrated degenerative enlargement of the proximal descending aorta immediately distal to the left subclavian artery, with dissection extending into the descending aorta. The aneurysm had enlarged from 54.9 mm at onset to 60 mm at the time of referral. The aortic diameter distal to the aneurysm measured 37 mm. The primary entry tear was located in the proximal descending thoracic aorta (**Fig. 1A**, white arrow).

Thoracic endovascular aortic repair (TEVAR) was considered unsuitable because of the enlargement of the ascending aorta (diameter: 42 mm). In addition, the distal extent of the arch aneurysm approached the level of the tracheal carina, often considered near the practical limit for distal anastomosis via median sternotomy; therefore, TAR with FET was selected to avoid a technically demanding deep distal anastomosis and to facilitate a more controlled proximal repair. We were concerned that TAR with FET alone would be insufficient to achieve favorable aortic remodeling owing to persistent retrograde perfusion of the FL. Therefore, we elected to perform TAR with adjunctive balloon fracture fenestration (BFF) to eliminate retrograde FL perfusion and promote distal sealing of the stent graft.

**Fig. 2:**
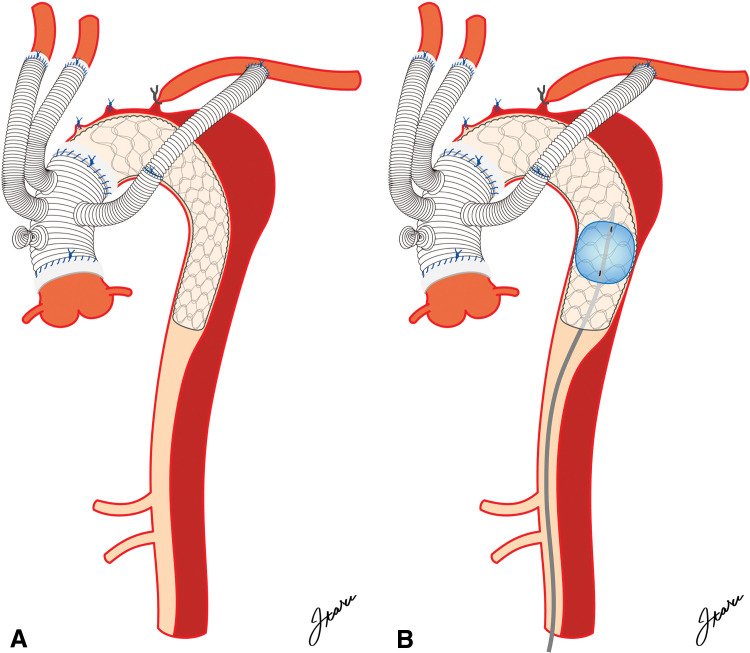
Schematic of aneurysm exclusion using balloon fracture fenestration after FET deployment. Schematic diagrams illustrating the aortic aneurysm exclusion technique after FET deployment, before and after balloon graft dilation (panels **A** and **B**, respectively). FET, frozen elephant trunk

TAR was performed using a zone 0 arch repair strategy, which included ascending aortic replacement, reconstruction of the arch vessels, and FET deployment from zone 0 into the descending aorta.^5)^ A FET graft measuring 39 mm in diameter (J Graft FROZENIX, 150 mm length; Japan Lifeline, Tokyo, Japan) was deployed (**Fig. 2A** and **Supplementary Video 1**). Graft diameter was determined from preoperative contrast-enhanced CT, corresponding to 110% of the proximal descending aortic diameter just distal to the arch aneurysm. The distal circulatory arrest time was 46 min, aortic cross-clamp time was 95 min, selective cerebral perfusion time was 115 min, and cardiopulmonary bypass time was 182 min.

After completion of TAR, a pigtail catheter was introduced into the FET via the right common femoral artery. Aortic anatomy and guidewire positioning within the true lumen were confirmed using intravascular US. Aortography demonstrated persistent endoleak into the arch aneurysm through the FL (**Supplementary Video 1**). A 0.035-inch extra-stiff guidewire (Lunderquist; Cook Medical, Bloomington, IN, USA) was then advanced through the outer catheter into the FET. The stent graft was dilated in the aorta distal to the aneurysm (37 mm in diameter) using a compliant endovascular balloon (40-mm Coda; Cook Medical) to fracture the chronic dissection flap (**Fig. 2B**). The balloon was inflated to 40 mm by injecting 40 mL of a 1:3 mixture of saline and contrast medium. Final aortography demonstrated complete exclusion of the aneurysm, with no residual endoleak.

**Fig. 3:**
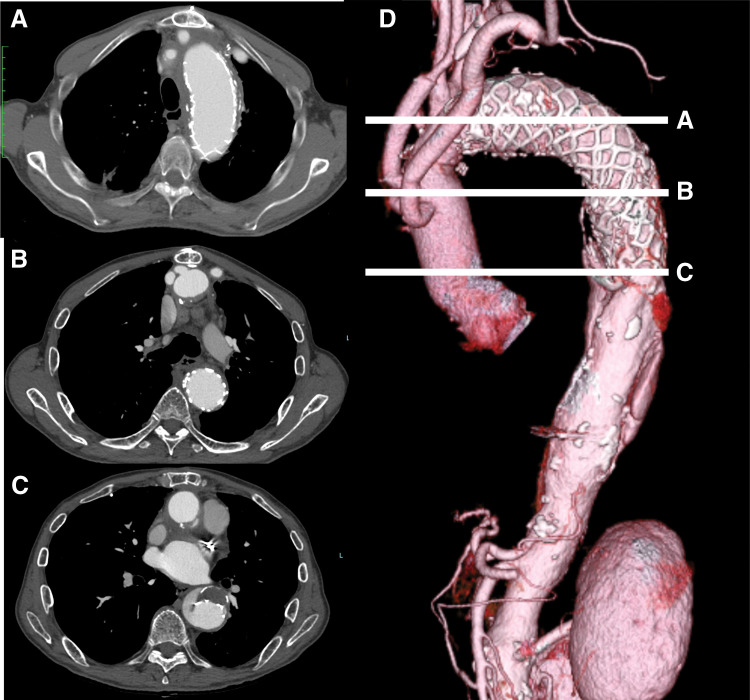
Postoperative CT demonstrating aneurysm exclusion and aortic remodeling. CT axial images at the levels of the aortic arch and proximal descending aorta, bronchial bifurcation, and left atrium at 6 months after surgery (panels **A**, **B**, and **C**, respectively). A 3D CT reconstruction at 6 months is shown in panel **D**. White lines A, B, and C in panel **D** correspond to the axial images in panels **A**, **B**, and **C**, respectively.

The postoperative course was uneventful. At 6 months, follow-up CT demonstrated a reduction in aneurysm diameter from 60 mm preoperatively to 53 mm, with an additional finding of distal stent graft-induced new entry (dSINE) in the descending aorta. Complete thrombosis of the aneurysm was observed, with no communication between the proximal and distal FLs (**Fig. 3**). During 3 years of follow-up, no endoleak or enlargement of the aortic arch aneurysm has been detected. The patient has experienced no aortic events and remains ambulatory, with regular outpatient follow-up.

## DISCUSSION

The novelty of this case lies in the concomitant application of BFF during TAR with FET, thereby converting a conventionally staged strategy into a single-stage repair. Performing BFF intraoperatively reinforced distal sealing and interrupted retrograde FL pressurization, enabling aneurysm exclusion within the same anesthetic. Although not intended as a routine adjunct to FET, this approach may represent a useful option in anatomically suitable patients—such as the present case with rapidly progressive aneurysmal enlargement—with concern for aneurysm rupture driven by persistent retrograde FL perfusion between staged procedures.

In open repair of descending thoracic and thoracoabdominal aortic aneurysms involving the distal arch, proximal aortic control can be difficult or hazardous; therefore, deep hypothermic circulatory arrest (DHCA) with an open proximal anastomosis is selected in some patients. Comparative studies have demonstrated similar early mortality rates between DHCA with open proximal anastomosis and distal arch clamping. However, DHCA has been associated with a greater respiratory burden, reflecting the increased invasiveness of open proximal anastomosis.^6)^ To avoid highly invasive single-stage repair and to address anatomically complex disease, staged strategies using the elephant trunk technique have been adopted.^7)^ In addition, staged repair for extensive thoracoabdominal aortic aneurysms has been reported to reduce the risk of spinal cord injury compared with single-stage repair.^7)^ Although acceptable outcomes have been reported, staged repair carries inherent risks of patient dropout between stages and interval aortic rupture.^8)^

The FET technique enables concomitant treatment of the descending aorta following arch replacement. However, persistent retrograde FL perfusion may maintain aneurysm pressurization, resulting in incomplete aneurysm exclusion and inadequate aortic remodeling.^2)^ When proximal entry closure is performed for chronic aortic dissection without adjunctive measures to promote FL occlusion, complete FL thrombosis may not be achieved in up to 50% of patients.^9)^ Treatment options to control FL flow after FET include open graft replacement, TEVAR alone, and TEVAR with adjunctive techniques such as the candy plug or balloon fracture fenestration.^2,10,11)^ Levack et al.^4)^ reported the use of adjunctive endovascular balloon fracture fenestration during TEVAR for subacute or chronic aortic dissection, in which a compliant balloon fully expands the stent graft and intentionally disrupts the dissection flap to control FL flow. The Knickerbocker technique, described by Rohlffs et al.,^11)^ employs a double-tapered tubular endograft with an asymmetric bulbous segment oriented toward the dissection membrane; balloon dilation of the expandable portion fenestrates and fractures the septum, allowing the graft to expand to the outer aortic wall and preventing retrograde perfusion of the thoracic FL proximal to the reno-visceral segment. We applied these endovascular concepts to TAR with FET. By combining TAR using FET with adjunctive balloon fracture fenestration, complete aneurysm exclusion was achieved in a single stage. This approach may mitigate a major limitation of staged repair, namely the risk of interval rupture while awaiting completion of the second-stage procedure.

A recognized concern with balloon fracture fenestration is the risk of dSINE. Levack et al.^4)^ reported visceral malperfusion due to dSINE in 1 of 49 patients who underwent ballooning along the stent graft, including its distal end, and noted that SINE cannot be entirely prevented because it may arise from multiple factors. As a technical modification, they proposed performing balloon dilation 3–4 cm proximal to the distal stent edge rather than directly expanding the distal end itself. In our case of an aneurysm associated with subacute TBAD (63 days after onset), postoperative CT demonstrated dSINE, which may have been related to reduced mobility of the dissection flap during the subacute phase. When dSINE is identified postoperatively, surveillance may be appropriate in asymptomatic patients with stable or decreasing aortic diameter and no evidence of ongoing FL pressurization or malperfusion. Reintervention—either additional TEVAR or open repair—should be considered if dSINE is accompanied by aneurysm enlargement, persistent or increasing FL perfusion, symptoms, malperfusion, or imaging findings suggestive of impending rupture. In some cases, a mismatch between the proximal FET diameter and the distal true-lumen diameter may necessitate the use of multiple endovascular devices to achieve adequate distal sealing.

This maneuver also carries a potential risk of iatrogenic aortic injury. Although no aortic rupture was reported in the series by Levack et al.^4)^ or Rohlffs et al.,^11)^ related septal-disruption strategies performed for aortic dissection from the acute to chronic phase (e.g., the Knickerbocker and STABILISE techniques) have reported rupture rates of approximately 2.5%–4.0% associated with balloon expansion at the distal end of the stent graft or within the bare-stent segment.^12–14)^ To mitigate this risk, balloon dilation should be limited to approximately the deployed stent-graft diameter and, consistent with general principles for SINE prevention, should be performed away from the distal stent edge.

Adjunctive BFF after TAR with FET may be considered in selected TBAD-associated arch or proximal descending aneurysms, particularly in the subacute and chronic phase, when intraoperative imaging demonstrates a patent FL with persistent retrograde FL perfusion into the aneurysm, suggesting ongoing pressurization despite FET deployment. Conversely, when intraoperative angiography shows no residual aneurysm filling, a delayed strategy with early postoperative imaging and staged endovascular intervention may be appropriate. Anatomical prerequisites include (1) a distal landing zone diameter smaller than the maximally expandable FET diameter (to permit distal sealing after ballooning), (2) aneurysm extent confined to the treatable thoracic segment covered by the chosen FET length (e.g., with a 150-mm FET, disease limited approximately to the level of the ninth thoracic vertebra,^15)^ and (3) a configuration in which balloon expansion can be performed within the stent-graft diameter and away from the distal stent edge. Traditional teaching in aortic endografting prohibits ballooning of acute aortic dissection due to the risk of aortic injury. The technique should be applied cautiously in acute dissections and individualized based on aortic morphology, FL patency or thrombosis, and overall operative risk. Long-term durability of this technique has not yet been established. To appropriately assess late aortic remodeling and detect potential aneurysm re-expansion due to FL reperfusion or progression of distal aortic dilatation, this patient has undergone contrast-enhanced CT at 1, 3, and 6 months, at 1 year postoperatively, and annually thereafter.

Placement of the distal end of the FET at the level of the tenth thoracic vertebra or lower may increase the risk of paraplegia related to spinal cord ischemia (SCI).^16)^ In the present case, the FET length was selected to limit distal stent coverage to approximately the T9 level. In patients with extensive FL patency requiring longer distal coverage, or in those in whom proximal entry closure is expected to induce widespread FL thrombosis, a staged strategy—consisting of TAR with or without FET followed by a planned second-stage TEVAR or open repair—may represent an appropriate alternative to balance aneurysm exclusion with the risk of spinal cord ischemia (SCI).

## CONCLUSIONS

Balloon fracture fenestration applied to TAR with FET may immediately eliminate retrograde FL perfusion, enhance distal sealing, and achieve durable aneurysm exclusion in carefully selected arch or proximal descending aneurysms associated with TBAD. This approach may mitigate a major limitation of staged repair, namely the risk of interval rupture while awaiting completion of the second-stage procedure.

## SUPPLEMENTARY MATERIAL

Supplementary Video 1 Preoperative and postoperative CT and operative footage.
